# Isolated Displaced Fracture of the Acromion: A Rare Case Report and the Consequence of Treatment by Open Reduction and Pin Fixation

**DOI:** 10.5812/atr.8762

**Published:** 2013-02-01

**Authors:** Seyed Abdolhossein Mehdi Nasab

**Affiliations:** 1 Musculoskeletal and Rehabilitation Research Center, Ahvaz Jundishapur University of Medical Sciences, Ahvaz, IR Iran

**Keywords:** Acromion, Scapula, Shoulder Pain

## Abstract

Displaced fracture of the acromion without associated shoulder injury occurs rarely. A 31-year-old gentleman presented with an isolated acromion fracture resulting from a direct trauma to the top of his left shoulder. Open reduction and internal fixation using multiple pins was performed and the fracture was closed without complications. The patient achieved excellent shoulder function and ranked as satisfied in the constant shoulder score almost 14 months following the surgery. Fixation with pins can provide adequate stability in this rare type of shoulder fracture.

## 1. Introduction

Fracture of the scapula occurs infrequently. Acromion is the lateral projection of the spine of scapula and fracture of this bone is a rare orthopedic condition as it accounts for only 8% of all scapular fractures (1). Most of these fractures have been associated with concomitant skeletal and soft tissue injuries of the shoulder (2, 3). With a significantly displaced fracture of the acromion, the normal function of the shoulder may be impaired or the sub acromial space compromised by compression of the rotator cuff, and the long head of the biceps tendon or deltoid muscle, causing impingement syndrome (4, 5). Open reduction and internal fixation is indicated when the fracture is associated with significant displacement. We are presenting a case with an isolated displaced fracture of the acromial base as well as the results of his operative treatment up to 14 months follow-up.

## 2. Case Report

**Figure 1:**
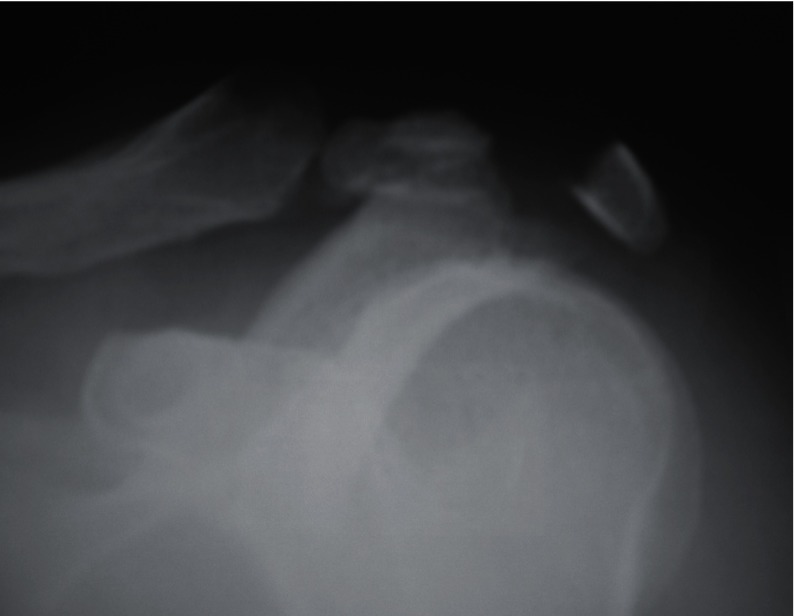
Anteroposterior Radiograph of the Shoulder Showing Displaced Fracture of the Acromial Base

**Figure 2:**
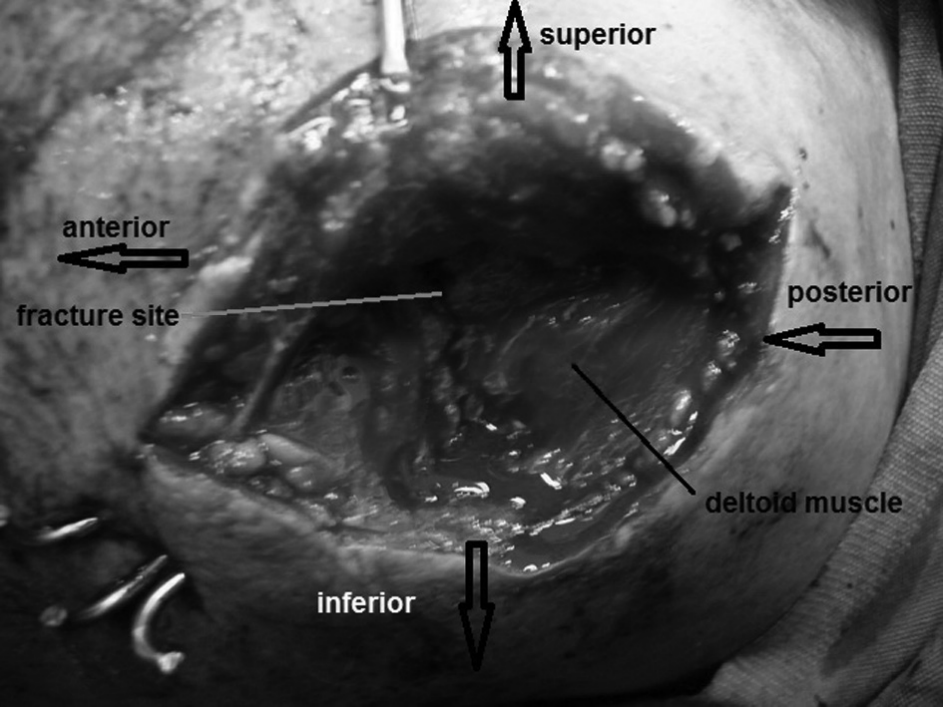
Intra Operative View Showed Fixation of Acromion with 3 Pins

**Figure 3:**
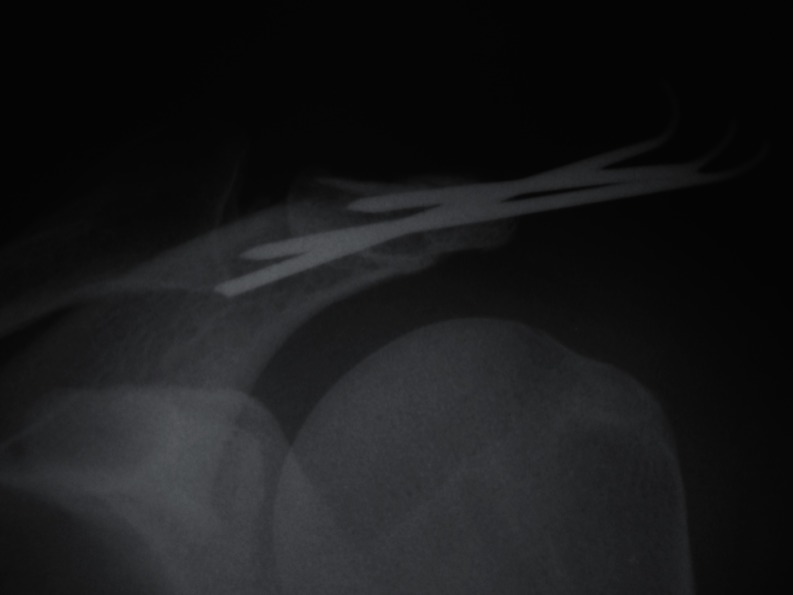
Postoperative Radiograph

**Figure 4:**
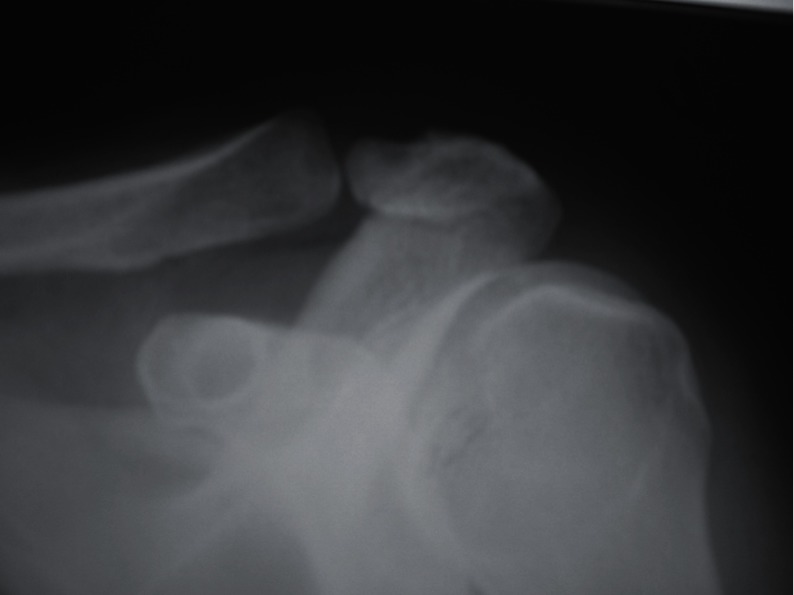
Radiograph of the Shoulder Revealed Complete Healing 14 Months Following the Treatment

A 31-year-old carpenter referred to our hospital with acute pain and swelling of his left shoulder following a direct trauma by dropping a heavyweight wooden block on the top of his shoulder. On examination there was swelling and tenderness over the lateral end of the clavicle and acromion. Neurological and vascular examinations of the upper limb were normal. Passive and active motion of the shoulder was painful and limited. The radiograph of the shoulder revealed a severely displaced fracture at the base of the acromion (Figure 1). Sixteen hours following the trauma, the patient was prepared for surgery. Under general anesthesia, through a superior approach, the fracture of the acromion was exposed and fixed with 3 pins (Figures 2 and 3). There was no deltoid or rotator cuff tearing. Following the operation, the shoulder was immobilized in a sling and swathe and daily dressing of the pins was recommended. Gentle active motion and progressive exercise of the arm and shoulder was initiated a week post-surgery. One of the pins extruded and was easily removed because of loosening 4 weeks following surgery. By ten weeks, and following a radiographic union of the fracture, other pins were removed. No pin site infection had been noted. The range of abduction was about 10º less in comparison to a normal contra lateral shoulder. Physiotherapy and strengthening exercises of the shoulder were continued until the maximal recovery was achieved 4 months later, and he could return to the pre-injury activity working level 6 months following treatment. At a final examination performed 14 months following the injury, the patient was satisfied and developed acceptable functional outcomes according to the shoulder constant score (Figure 4).

## 3. Discussion

Fractures of the scapula are infrequent with acromion fracture being an even rarer injury. Scapular fractures represent 3-5% of all shoulder injuries and 0.4 - 1% of all fractures (2, 4). Acromial fractures account for only 8% of all scapular fractures (1). Kuhn et al. in a review of 27 fractures of the acromion during a 15-year period defined five distinct types that were classified into three groups. Stress fractures, Type I with minimal displacement and Type IA avulsion fractures, which heal rapidly. Type IB fractures result from direct trauma, with minimal displacement. Type II displaced fractures that do not reduce the sub acromial space are treated by a non-operative technique; however, a type III with inferior displacement and with sub acromial space involvement requires surgical treatment (6). Scapular fractures have been classified into three types including acromion, coracoids and body fractures. This fracture is usually caused by a direct trauma or a violent contraction of the surrounding muscles, and may be isolated or can also arise with associated shoulder injuries. With respect to the isolated acromion fracture, there are a few case reports in the literature. In those patients, the causes of the fracture has been by a superior displacement of the humeral head, stress fracture or evulsions by a single violent muscle contraction of deltoid or sub scapularis tendon or repetitive sub maximal load to the shoulder (7-10). This fracture can be managed by non-operative or surgical means depending on the initial displacement. For fixation of displaced acromion fracture, a variety of techniques including tension band wiring, screw, reconstruction plate, and kirschner wire have been advocated (6, 8). In our case, despite significant displacement of acromion fracture, no other skeletal or soft tissue injury was noted. A minimal incision on the acromion with direct muscle splitting approach was a safe and easy technique to expose and reduce the fracture site. One pin extruded and had to be removed due to loosening by 4 weeks; however, no infection had occurred. The other pins were stable and removed 6 weeks following the surgery. Loosening and pin tract infection is a complication of the smooth pin fixation. To prevent this complication and potential loss of fixation or pin site infection, insert the treaded pin, cut the end pin beneath the skin, and achieve fixation by screw. Another modality such as a screw or narrow reconstruction plate could be used. Displaced fracture of the base of acromion may have resulted from direct trauma without associated shoulder injuries. The use of open reduction and multiple pin fixations worked out well for the young man with this fracture.

## References

[1] Goss TP (1996). The scapula: coracoid, acromial, and avulsion fractures.. Am J Orthop (Belle Mead NJ)..

[2] Butters KP, Beaty JH, Rockwood CA, James R, Kasser MD (2010). Fractures of the scapula. Rockwood and Wilkins' Fractures in Children: Text Plus Integrated Content Website..

[3] Lim KE, Wang CR, Chin KC, Chen CJ, Tsai CC, Bullard MJ (1996). Concomitant fracture of the coracoid and acromion after direct shoulder trauma.. J Orthop Trauma..

[4] Ada JR, Miller ME (1991). Scapular fractures. Analysis of 113 cases.. Clin Orthop Relat Res..

[5] Crenshaw AH, Perez EA, Campbell WC, Canale ST, Beaty JH (2008). Fractures of the shoulder. Campbell's operative orthopaedics..

[6] Kuhn JE, Blasier RB, Carpenter JE (1994). Fractures of the acromion process: a proposed classification system.. J Orthop Trauma..

[7] Gorczyca JT, Davis RT, Hartford JM, Brindle TJ (2001). Open reduction internal fixation after displacement of a previously nondisplaced acromial fracture in a multiply injured patient: case report and review of literature.. J Orthop Trauma..

[8] Madhavan P, Buckingham R, Stableforth PG (1994). Avulsion injury of the subscapularis tendon associated with fracture of the acromion.. Injury..

[9] Stoll M, Lill H, Wuttke M, Josten C (2001). [Fracture of the acromion. Diagnosis-treatment strategy-outcome].. Unfallchirurg..

[10] Weber D, Sadri H, Hoffmeyer P (2000). Isolated fracture of the posterior angle of the acromion: a case report.. J Shoulder Elbow Surg..

